# The fisheries and aquaculture sector in Latin America: Exports to East Asia and production

**DOI:** 10.1371/journal.pone.0267862

**Published:** 2022-07-26

**Authors:** Rebeca De las Mercedez Jaime Muñiz, Juan Antonio Jimber del Río, Francisco Javier Jiménez Beltrán, Paúl Vera Gilces

**Affiliations:** 1 Department of Research, Ecotec University, Guayaquil, Ecuador; 2 Universidad de Cordoba, Córdoba, Spain; 3 Universidad Ecotec, Guayaquil, Ecuador; 4 ESPAE Graduate School of Management, ESPOL Polytechnic University Campus Las Peñas, Guayaquil, Ecuador; 5 Trade Data Lab, Guayaquil, Ecuador; Universidad Nacional Autonoma de Nicaragua Leon, NICARAGUA

## Abstract

The Asian giant has agitated foreign trade in Latin America due to easy access to this market, tariff policies, and international agreements. In the face of a globalized world, in the last decade fisheries and aquaculture entrepreneurs of Latin American countries have taken on the challenge of getting their products known at International Fisheries and Aquaculture Exhibition fairs in Asian countries, which has generated an increase in the exportation of their products. Fishing and aquaculture exports are a variable of the economy of each country reflected in the gross domestic product and foreign exchange results. The present research work analyzed the fishing and aquaculture sector through the first difference Generalized Method of Moments estimator to establish the behavior of exports from Latin American countries to Asia, the production of each Latin American country, and the subsequent effect on the economic growth of the fishing and aquaculture sector during the period 2012–2019. The findings of this article suggest the significant positive relationship between output and economic growth, and a non-significant negative relationship between exports to East Asia and the economy.

## Introduction

The Exclusive Economic Zones (EEZ) of the coastal countries of Latin America extend to the southwestern Atlantic, the eastern Pacific, the southern oceans, and the Caribbean Sea. Historically, the region has contributed more than 15% of world marine fish production, although, most of it has been concentrated in fishmeal produced by massive catches of small pelagic fish in upwelling areas off Peru and Chile [1]. However, in recent decades, Latin American fishery production has not only increased on an ongoing basis but it has also diversified, shifting its focus towards fish production for human consumption (rather than fishmeal), and trading with the United States, the European Union, and Japan [2]. As the new millennium progressed, Latin America became one of the core net exporting regions, reporting in 2006 a surplus of US$ 8 billion in the trade of fish and fishery products [3]. However, several large countries in Latin America have also experienced a growing domestic demand for seafood, and regional fisheries development appears to have been motivated by foreign exchange earnings rather than food security [2].

The fishing industry contributes around 4.5% of the Latin American Gross Domestic Product (GDP). It is worth mentioning that fish constitutes a fundamental part of the regional diet. Latin America is bound to the ocean, nearly 275 million people work in fishing or aquaculture activities; in 2012, around US$15 billion of the regional contribution to GDP came from the ocean, being a rich area yet to be explored and researched, and less developed than agriculture [4].

In context, the value chains in Latin America and the relationship with the Asia Pacific have deepened in the last two decades, especially with the creation of new trade agreements. It is noteworthy that 20% of the added value is exported to the Asia Pacific: China, the Association of Southeast Asian Nations, Japan, and the Republic of Korea [5]. Latin American exports to Asia could grow by almost 27% with the reduction of tariffs, transport, and logistics since the Asian continent is considered the second-largest trading partner. In the last two decades, the share of Asia in Latin America tripled from 9% in 2000 to 26% in 2018, and it is estimated that the Asian demand for food will reach 3.5 billion people before 2030 [6].

Many Latin American developing countries should take advantage of marine resources considering the potential that foreign trade offers in this sector. There are several studies employing economic models based on global seafood supply and demand that can be used to analyze trends and the effect of such opportunities, in a way that both developing and developed countries can be informed about the importance and urgency of improving capture fisheries and aquaculture management to ensure that the demand for seafood is met in an environmentally and economically sustainable manner [7]. Fishing industries provide vital food for subsistence throughout the world; the challenge is to meet the demands while protecting the resources for future generations. For years, the governments of the WTO member countries have subsidized fishing through a negotiation program that allows a sustainable balance in fish production and the conservation of the marine ecosystem [8].

According to the literature and research reviewed, there is no evidence of any research conducted with direct relation to the one proposed in this study, which seeks to analyze the fishing and aquaculture sector in Latin America through the first difference Generalized Method of Moments estimator for autoregressive linear regression models estimated from short panels in the presence of unobserved individual-specific time-invariant effects. This model provides information to establish the behavior of exports from Latin American countries to Asia and the production of each Latin American country with its effect on the GDP of the fishing and aquaculture sector. The indicators of the comparative study of exports from Latin American countries to East Asia cover the period from 2012 to 2019. Therefore, the importance of the research topic and its validity are justified.

## Theoretical framework

Export growth is generally considered the main factor indicating output and employment growth in a country. This assumption is called export-led growth (ELG). The ELG literature is quite extensive, and Ullah, Zaman, Farooq, and Javid [9] provide empirical evidence from recent time-series by reporting a positive impact of export expansion on the economic growth in the Pakistani economy during the period 1970–2008. Regarding panel data techniques, Pazim, Hanim, & Fadzim [10] find no support for the ELG hypothesis for three Asian economies during the period 1985–2002. Within a neoclassical framework, Bahmani-Oskooee, Economidou, and Goswani [11] find weak evidence for the ELG hypothesis for 61 developing countries during the period 1960–1999.

Numerous publications have documented the relationship between exports and economic growth, but not many of them on sectoral exports and economic growth. Duc and Tram [12] examine the relationship between the evidence of Vietnamese fishery exports with GDP from 1997 to 2008. However, the effects of fishery exports on economic growth have not yet been thoroughly studied with an econometric approach. Descriptive and time-series analyses in this study present a positive effect of fishery exports on Vietnamese economic growth in the long run. The modern econometric approach with stationary and cointegration tests and vector error correction models used in this study also helped the authors to forecast the persistence of the effects of fishery exports on Vietnamese GDP despite different seasonal trade phases. For the long-term estimate, a two-fold increase in the value of its fishery exports would raise GDP by 7%. This has great economic significance in the development process of sectoral exports. On the other hand, Vietnamese fishery exports would increase by 5.2%, representing a GDP growth of 10%.

To explore the causal relationship between fish export growth and economic growth for 23 Small Island Developing States (SIDS) over the period 1989–2002, Jaunky [13] performed several panel unit root and cointegration tests. The results showed strong evidence of a long-term relationship between fish export growth and economic growth. In addition, the Blundell-Bond system GMM method is used to perform a Granger-type causality test within a vector autoregressive (VAR) panel data frame. The long-term estimate of the impact of fish export growth on economic growth is calculated using the fully modified ordinary least squares (FMOLS) method and is found to be 0.01. These results show that fish exports represent a means for SIDS to maintain their long-term economic growth and their importance in managing their fisheries.

A similar study was proposed by Béné [14], which analyzed fishery exports and GDP using panel data. This study was conducted on 47 sub-Saharan African countries during the period 1990–2005. The author considered a range of eight national development indicators that encapsulate both the economy and well-being of sub-Saharan countries over the last decade and that are correlated with four indicators that reflect the country-specific importance of fish trade, and the industrial and small-scale fishing in the sub-Saharan African economy. The results obtained by the author suggest that there is no relationship between the study variables.

According to Jawaid, Siddiqui, Atiq, and Azhar [15], who first attempted to explore the relationship between fish exports and the economic growth of Pakistan using annual time series data for the period 1974–2013, where the error correction model reveals that there is no immediate or short-term relationship between fish exports and economic growth, the long-term estimates of the model suggest that as fish exports increase by 1%, the GDP of Pakistan increases by 0.29%.

Fishery exports, according to Cunningham [16], Schmidt [17] and the FAO [18], may act as a growth engine for developing countries endowed with vast fish resources. Furthermore, earnings from fishery exports can contribute to economic growth in developing countries by providing a significant source of cash income [19].

Fishery exports not only contribute indirectly to economic development through the generation of new jobs but also increased revenues for the sector, and the secondary flow on effects such as migrant workers who transfer remittances to their families. Furthermore, most fishery exports have strong backward linkages with other sectors, both in terms of primary commodities and value-added products. However, there is a lack of empirical studies on a clear relationship between Latin American fishery exports and economic growth, even though incumbent governments have tried in practice to promote the growth of fishery exports to boost the economy.

## Materials and methods

### Data source and variables

This section presents the data obtained from the study of exports in the economic growth of 16 Latin American countries (Argentina, Brazil, Chile, Colombia, Costa Rica, Ecuador, El Salvador, Guatemala, Honduras, Mexico, Nicaragua, Panama, Paraguay, Peru, Dominican Republic, Uruguay), whose final destination is East Asia (China, Hong Kong, South Korea, Japan). Production data for the study period of the Latin American countries in the fishing and aquaculture sector was also obtained; the data set will be organized according to the variables, through the simple linear regression model in case there is any relationship between the variable exports and GDP (such as production and GDP), to determine the growth or contribution of this economic sector to GDP. This research analyzes the quantitative data that allows explaining the behavior of the data obtained, as well as the policies, agreements, norms, and regulations related to this sector that will allow the extraction of the main ideas to reach the conclusion.

For the dependent variable GDP of Latin American countries, information was obtained from ECLAC-CEPALSTAT through statistics and economic indicators in annual dollars per economic activity at current prices. To obtain the independent variables export and production of the fishing and aquaculture sector, the tariff headings corresponding to the fishing sector in Latin America were reviewed; the information was compiled from the Trade Map–International Trade Statistics database, and Un Comtrade.

### Econometric model

#### First difference GMM estimator

First, the GMM approach in first differences is established. To simplify, an AR(1) model with unobserved individual specific effects was considered

$$y_{it}=\alpha y_{i,t-1}+\gamma_{i}+\delta_{it}|\alpha |<1$$ (1)

for i = 1,…, N y t = 2,…, T, where *γ_i_*+*δ_it_* = *μ_it_* has the standard error component structure

$$E|\gamma_{i}|=0\;E|\delta_{it}|=0\;E|\gamma_{i}\delta_{it}|=0\;for\;i=1,\ldots ,N\;y\;t=2,\ldots ,T.$$ (2)

Transient errors are assumed to be serially uncorrelated

$$E|\delta_{it}\delta_{is}|=0\;para\;i=1,\ldots ,N\;y\;s\neq t$$ (3)

and that the initial conditions yi1 are predetermined.


$$E|y_{i1}\delta_{it}|=0\;para\;i=1,\ldots ,N\;y\;t=2,\ldots ,T.$$ (4)

Together, these assumptions imply the following moment constraints m = 0.5(T − 1)(T − 2)

$$E|y_{i,t-s}\Delta \delta_{it}|=0\;for\;t=3,\ldots ,T\;y\;s\geq 2$$ (5)

which can be more compactly written as

$$E(\dot{Z_{i}\Delta \delta_{i}})$$ (6)

where Zi is the matrix (T − 2) × m given by

$$Z_{i}=\left[\begin{array}{ccc} y_{i1} & \cdots & 0\\ \vdots & \ddots & \vdots \\ 0 & \cdots & y_{i,T-2} \end{array}\right]$$ (7)

and Δ*δ_i_* is the vector (T − 2) (Δ*δ_i_*3, Δ*δ_i_*4,…, Δ*δ_i_*T) 0. These are the moment restrictions exploited by the standard linear first-difference GMM estimator, which implies the use of lagged levels with date t − 2 and earlier as instruments for the first-difference equations. This produces a consistent estimator of α when N → ∞ with fixed T.

Based on the aforementioned, the joint analysis of the two variables in relation to the GDP, through the panel data model, will indicate the variables corresponding to the evolution and incidence of the production of Latin America and the exports of the fishing and aquaculture sector from Latin America to East Asia, and their impact on GDP. A panel data model was used in order to establish the group effect of both independent variables on economic growth, leaving the model as follows:

$$LPIB=\beta_{0}+\beta_{EXPAQUA}LEXPAQUA+\beta_{PROD}LPROD+\varepsilon$$ (8)

Where:

- LPIB: Economic growth
- LEXPAQUA: Fisheries and aquaculture exports in East Asian countries
- LPROD: Production of the fishing and aquaculture sector

The panel data model was used due to the characteristics of the data, by considering both cross-sectional and longitudinal data after analyzing 16 Latin American countries during the period 2012–2019.

## Results

For this research, an estimate was made using the GMM estimator. The validity of the model specification was also tested using the Sargan test, used for testing over-identifying restrictions in a statistical model, and the Arellano-Bond test was used to verify if the error terms of the regression are correlated in second-order series (set of instruments orthogonal to the estimated residuals). Both tests are shown in Table 1. Another concern regarding the validity of the presented model refers to the number of instruments used in the regression. Authors such as Forbes [20] have mentioned that the specification tests are vulnerable to the number of instruments used for the total number of observations. The instruments can be used when performing dynamic panel data analysis, especially when GMM methods are applied in many ways. The most common approach is to use all the possible sets of available regression tools. However, following the critique of Roodman [21], this approach can generate biases in the resulting estimates. To address this, Roodman presents some ways to avoid bias when using the instrument matrix. In doing so, the number of lags used as instruments in each regression is restricted to one for default variables and up to two for endogenous variables. Therefore, it is less likely that the estimates in this study will be biased.

**Table 1:** Results of the GMM model.

GDP	Coefficient	Standar Error	z	Value p
GDP				
L1	0,2027	0,0873	2,32	0,02
LPAP				
…	1,1842	0,6527	1,81	0,07
L1	-0,6874	0,6375	-1,08	0,28
EXP				
…	-0,0483	0,0623	-0,78	0,43
L1	-0,1498	0,0583	-2,57	0,01
Year 2	3,8608	0,3331	11,59	0
Year 4	1,3857	0,3713	3,73	0
Year 5	0,2877	0,3327	0,86	0,387
Year 6	1,3358	0,4051	3,3	0
Year 7	0,0517	0,3566	0,15	0,885
Year 8	0,6583	0,4329	1,52	0,128
Cons	15,8441	4,5922	3,45	0,01
Sargan	0,17			
AR (1)	0,0367			
AR (2)	0,4981			

Note: P-values for Sargan and AR are presented. Sargan is the p-value of a constraint overidentification test, which tests the general validity of the instruments. AR(1) and AR(2) are the p-value of a first and second order serial correlation test.

Exports were modeled as endogenous since there is a strong dependence on GDP measured by the expenditure approach (includes exports). On the other hand, fishing and aquaculture production is modeled exogenously because it is not related to GDP. Finally, the use of instruments such as the fixed effects of years, which control the macroeconomic conditions of the countries in this study, was taken into consideration.

Table 1 shows the results of the first difference GMM estimator, it can be observed that the first lag of the dependent variable is significant, indicating that the past results of the GDP and the model in general influence the present, justifying the use of the dynamic panel. Taking into account what was previously described, the sign of the coefficients, the aquaculture, and fishing production was later interpreted. A positive relationship with economic growth was observed, this variable being significant at 10%. While a significant relationship between aquaculture and fisheries exports to East Asia with GDP could not be established despite having a negative sign. Finally, the Sargan test gave a p-value greater than 10%, validating the model specification, and the Arellano-Bond test yielded a significant p-value in the first lag and a non-significant p-value in the second lag, thus, suggesting the autocorrelation of the first-order autocorrelation and the non-existence of second-order autocorrelation.

## Discussion

The results suggest a positive and statistically significant relationship between production and GDP. These empirical findings are consistent with Ferreira, Saurel, e Silva, Nunes, and Vázquez [22], who indicate that many countries rely on revenue from marine wealth to boost purchasing power. On the other hand, Al-Ghadban [23] and Al-Jamali et al. [24] claim that fish trade enhances competitive market forms and increases GDP.

However, a negative and non-significant relationship was found between exports and GDP. These results differ from what was found in the literature review since authors such as Béné [14] Duc & Tram [12], Jaunky [13], Jawaid, Siddiqui, Atiq & Azhar [15], established that aquaculture and fisheries exports positively influence economic growth. This, in the opinion of the authors, can be explained since the present work focused on the variable exports directed towards East Asia, and if global exports had been considered, the sign found would possibly have been positive.

## Conclusion

Fishing is the most common profession in Latin America and constitutes an important source of income through production and exports, which boosts the economy. Fishery products are one of the most important traded food products. In this article, an attempt is made to empirically identify the relationship between fisheries and aquaculture production and exports with economic growth in Latin America by using annual time series data for the period 2012–2019. The findings of this article suggest a significant positive relationship between production and economic growth, and a negative and non-significant relationship between exports to East Asia and GDP, through the first difference GMM estimator. To test the validity of the model, the Sargan and the Arellano-Bond tests were applied, which gave solidity to the initial results.

The results of this study highlight the importance of fishing in contributing to GDP growth. Since it is an important source of income for coastal inhabitants, this has caused the capture of fish to be faster than the reproduction of the species. UN experts have warned that the oceans will be empty of fish by 2050 if appropriate action is not taken [15]. The effects of climate change on water bodies affect salinity levels and therefore population growth, in turn affecting the efficiency of production systems [25]. A decrease of up to 40% of fishing is expected due to climate change in tropical regions, causing repercussions on primary productivity, mainly affecting fish populations that carry out their reproduction in reefs and estuaries [26] According to Brander y Taylor [27], overexploitation occurs when the marine resource is easily accessible and the catch exceeds the limit. in such a way that there are no fish left in the ocean to reproduce and repopulate the fish population. One way to address this problem at the national level is to grant exclusive use rights that allow fishermen to catch a specific amount of fish in a given period of time. In addition, the existing laboratory infrastructure should be improved. Laboratories must provide certification reports and tests according to international standards that are acceptable to the countries that have the highest global demand. The existing fleet of fishing boats needs to be modified as it will help the inhabitants to fish efficiently. More importantly, the fish can be raised by maintaining aquaculture to meet the growing demand and prevent depletion. Aquaculture should be practiced and maintained regularly under controlled conditions to improve fish production and protection from predators.
